# Coronary Atherosclerotic Plaque Vulnerability Rather than Stenosis Predisposes to Non-ST Elevation Acute Coronary Syndromes

**DOI:** 10.1155/2019/2642740

**Published:** 2019-03-11

**Authors:** Mohamed Laimoud, Farouk Faris, Helmy Elghawaby

**Affiliations:** Critical Care Medicine Department, Kasr Alainy Hospitals, Cairo University, Egypt

## Abstract

**Background:**

Non-ST elevation acute coronary syndromes (NSTE-ACS) may arise from moderately stenosed atherosclerotic lesions that suddenly undergo transformation to vulnerable plaques complicated by rupture and thrombosis.

**Objective:**

Assessment and tissue characterization of the coronary atherosclerotic lesions among NSTE-ACS patients compared to those with stable angina.

**Methodology:**

Evaluation of IVUS studies of 312 coronary lesions was done by 2 different experienced IVUS readers, 216 lesions in 66 patients with NSTE-ACS (group I) versus 96 lesions in 50 patients with stable angina (group II). Characterization of coronary plaques structure was done using colored-coded iMap technique.

**Results:**

The Syntax score was significantly higher in group I compared to group II (18.7 ± 7.8 vs. 8.07 ± 2.5, *p*=0.001). Body mass index (BMI) was significantly higher in group II while triglycerides levels were higher in group I (*P*=0.01 & *P*=0.04, respectively). History of previous MI and PCI was significantly higher in group I (*P*=0.016 & *P*=0.001, respectively). The coronary lesions of NSTE-ACS patients had less vessel area (9.86 ± 3.8 vs 11.36 ± 2.9, *p*=0.001), stenosis percentage (54.7 ± 14.9% vs 68.6 ± 8.7%, *p*=0.001), and plaque burden (54.4 ± 14.7 vs 67.8 ± 9.8, *p*=0.001) with negative remodeling index (0.95 ± 20 vs 1.02 ± 0.14, *p*=0.008) compared to the stable angina group. On the other hand, they had more lipid content (21.8 ± 7.03% vs 7.26 ± 3.47%, *p*=0.001), necrotic core (18.08 ± 10.19% vs 15.83 ± 4.9%, *p*=0.02), and calcifications (10.4 ± 5.2% vs 4.19 ± 3.29%, *p*=0.001) while less fibrosis (51.67 ± 7.07% vs 70.37 ± 11.7%, *p*=0.001) compared to the stable angina patients. Syntax score and core composition especially calcification and lipid content were significant predictors to NSTE-ACS.

**Conclusions:**

The vulnerability rather than the stenotic severity is the most important factor that predisposes to non-ST segment elevation acute coronary syndromes. The vulnerability is related to the lesion characteristics especially lipidic core and calcification while lesion fibrosis favours lesion stability.

## 1. Introduction

Acute coronary syndromes frequently arise from erosion of vulnerable plaques and subsequent thrombosis [1, 2] while stable coronary atherosclerotic plaques progress to stenosis causing stable angina [3].

Previous studies have noted that histologic thumbprints of these vulnerable plaques were characterized by thin-capped atheroma with a lipid-rich core, few smooth muscle cells, numerous macrophages, adventitial inflammation, and positive remodeling [4–6].

The main challenge faced was that ACS often arises from lesions with only mild to moderate stenosis. Accordingly, disclosure of potentially vulnerable plaques may promote prevention of cardiovascular events [7, 8].

## 2. Aim of Work

Our objective was to study the coronary atherosclerotic lesion characteristics that predict the occurrence of non-ST segment elevation acute coronary syndromes compared to those with stable angina.

## 3. Patients and Methods

We did this cross-sectional study on 116 patients (50 patients with stable angina and 66 patients with non-ST segment elevation acute coronary syndromes) subjected to coronary catheterization between the periods from January 2017 to January 2018.

Stable angina is defined as a clinical syndrome characterized by discomfort in the chest, jaw, shoulder, back, or arm. It is typically aggravated by exertion or emotional stress and relieved by nitroglycerin with no change in character for sixty days. Angina usually occurs in patients with CAD involving ≥1 large epicardial artery [9].

Chest pain in absence of persistent ST elevation with elevated cardiac biomarkers of necrosis is suggestive of NSTE-ACS [10].

Informed consents were obtained from all individual participants included in this study. The study had been approved by the institutional research ethical committee.

Patients with acute ST elevation myocardial infarction (STEMI), previous coronary artery bypass grafting (CABG), renal impairment, and thrombocytopenia were excluded.

Coronary angiography and interventions were done using Philips (CV20, 2011- Netherland) and Siemens (Axiom Artis DFC 35875) with 15 frames per second (fps) imaging speed.

Three hundred twelve coronary lesions were assessed by IVUS during diagnostic angiography, 216 lesions in 66 patients with NSTE-ACS (group I) versus 96 lesions in 50 patients with stable angina (group II).

### 3.1. Intravascular Ultrasound Imaging Protocol and Analysis

We used iLab™ Ultrasound Imaging System (90539386-01A, 2009-Boston Scientific Inc., USA) to get IVUS runs, after administration of 200 mcg intracoronary nitroglycerin, using a 40 MHz 6F compatible catheter (Atlantis SR Pro).

Image acquisition was done through retrograde-automated transducer pullback at 0.5 mm/second. The pullback started from at least 10 mm distal to the studied lesions as the distal reference segment was defined as the site with the largest lumen distal to a stenosis but within the same segment [11].

Based on images depicted during pullback of the transducer, the lesion was defined as the image slice with the smallest lumen cross-sectional area [11].

The measurements were taken according to the American College of Cardiology guidelines, and reporting was done by two experienced IVUS readers [11].

Lumenal and external elastic membrane (EEM) cross-sectional areas (CSAs) were measured for each 1 mm of axial length, and then, plaque plus media (P&M) CSA was calculated by EEM CSA minus lumen CSA.

Plaque burden was calculated by P&M CSA divided by EEM CSA. The lesion was considered significant when percent area stenosis >70%.

The distal and proximal vessel reference segments were the most apparently normal segments within 10 mm distal and proximal to the coronary lesion.

The remodeling index (RI) was calculated by EEM CSA divided by the mean reference CSA [12].

The colored-coded iMap technique was used to get the characteristics of coronary plaques structure.

### 3.2. Statistical Analysis

Precoded data were entered on the computer using “Microsoft Office Excel Software” program (2010) for Windows. Data were then transferred to the Statistical Package of Social Science Software program, version 21 (SPSS), to be statistically analyzed.

Data were summarized using mean, standard deviation, median, and interquartile range for quantitative variables and frequency and percentage for qualitative ones.

Comparison between groups was performed using the independent sample *t*-test or one-way ANOVA with Tukey's post hoc test for quantitative variables and the chi-squared test or Fisher's exact test for qualitative ones.

Univariate regression analysis had been used to determine potential predictors of ACS.

*P* values less than 0.05 were considered statistically significant and less than 0.01 were considered highly significant. Graphs were used to illustrate some information.

## 4. Results

### 4.1. Demographic and Clinical Data

Body mass index (BMI) was significantly higher in the stable angina (SA) group while the triglyceride level was higher in the NSTE-ACS group.

History of previous MI and PCI was significantly higher in the NSTE-ACS group (Table 1).

### 4.2. Angiographic Data

The study involved 312 coronary lesions, and the culprit lesions were 134 for which stenting was done. LM lesions were higher in the NSTE-ACS patients compared to SA patients. The Syntax score was significantly higher in the NSTE-ACS group compared to the SA group (Table 2).

### 4.3. Intravascular Ultrasound (IVUS) Data

Notably, the coronary lesions of NSTE-ACS patients had significantly less vessel area with negative remodeling index compared to those with the SA group. Also, the plaque burden and percent area stenosis were significantly smaller than those of the SA group (Table 3).

**Figure 1:**
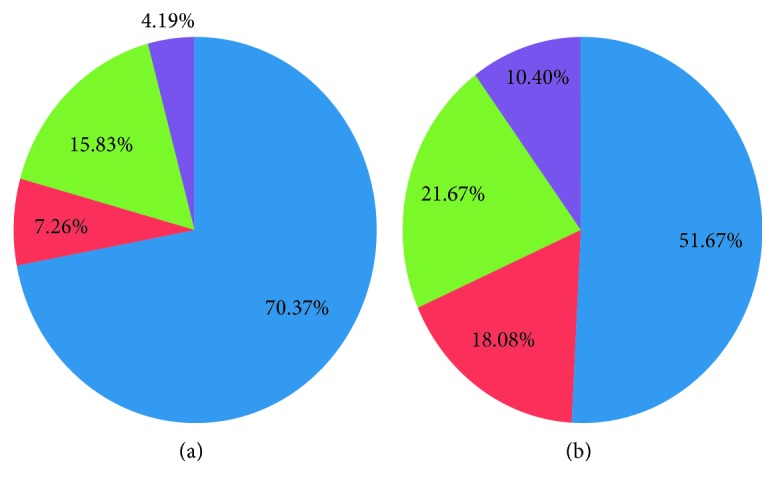
Coronary lesions component. (a) Stable angina patients. (b) ACS patients. Blue = fibrotic component. Green = lipidic component. Violet = calcium component. Red = necrotic component.

**Figure 2:**
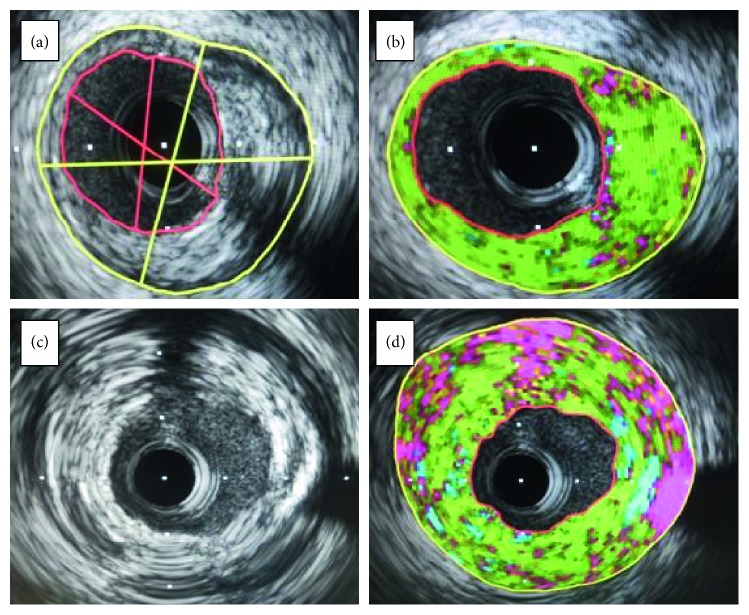
IVUS runs of LAD lesions. (a) Greyscale run of SA patient. (b) iMAP study of lesion in (a). (c) Greyscale run of NSTE-ACS patient. (d) iMAP study of lesion in (c). Green = fibrous component. Yellow = lipid component. Red = necrotic component. Blue = dense calcium.

Regarding lesion characteristics, NSTE-ACS group lesions had significantly more lipidic content, necrotic core, and calcifications with less fibrosis compared to the stable angina patients (Table 4; Figures 1 and 2).

### 4.4. IVUS Predictors of Acute Coronary Syndromes

**Figure 3:**
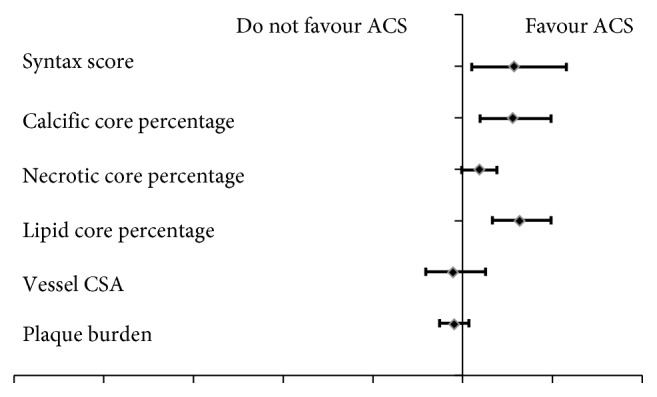
Predictors of NSTE-ACS.

Univariate regression analysis had been used to determine potential predictors of NSTE-ACS and had showed that the Syntax score and core composition especially calcification and lipid composition were significant predictors (Table 5 & Figure 3).

## 5. Discussion

Plaque destabilization is a biomechanical phenomenon depending on complex interactions between applied shear stresses, resulting in reactive oxygen species production and inflammation, blood laminar or turbulent flow characteristics, coronary lesion structural features, and biological processes that determine mechanical strength [1, 13–17].

Plaque rupture refers to transmural fissuring of the atheroma fibrous cap and exposure of the underlying necrotic core with its proinflammatory and thrombogenic activities to circulating blood. It is the commonest form of plaque destabilization. The ruptured plaque usually has the features of a thin cap fibroatheroma [1, 18–20]; plaque erosion describes a histologically seen thrombus on the endothelial layer of a nonruptured coronary plaque [21]. Also, lesions with calcified nodules constitute another rupture-prone plaque because they had been found in some culprit lesions, exerting disruptive effects on plaque integrity [18, 19].

Our study used IVUS to assess the atherosclerotic burden and the plaque vulnerability in culprit and nonculprit vessels during cardiac catheterization of patients with stable angina and NSTE-ACS. Both groups had similar risk factors of atherosclerosis except for hypertriglyceridaemia and BMI. NSTE-ACS patients had moderately stenosed coronary lesions with less plaque burden but negative remodeling index as compared to patients with stable angina.

Our results were similar to those of Ghaffari et al. [22] which concluded that most of the lesions leading to myocardial infarction have a diameter stenosis of at least 50%. Also, Giroud et al [23] studied 184 consecutive angiograms of 92 patients who had underwent coronary angiography both before and after acute myocardial infarction and concluded that the severity of the narrowing on the first angiogram was a poor predictor of subsequent infarctions. Similarly, Little et al. [24] monitored 29 patients after coronary angiography until they presented with MI and concluded that the majority of the cases of MI arose from nonsignificant coronary stenosis.

Our study showed that NSTE-ACS patients had a higher ratio of lipidic content, dense deep calcium, and necrotic core components, while less fibrotic content compared to stable angina patients. This could explain the occurrence of ACS despite having moderately stenosed coronary lesions with less plaque burden. The lesion characteristics were similar to those in Nakamura et al.'s study [25] which concluded that ACS patients showed significantly higher ratio of dense calcium and necrotic core plaque compared with SA patients.

The PROSPECT (Providing Regional Observations to Study Predictors of Events in the Coronary Tree) study [26] confirmed the importance of shallow necrotic cores as one of the most significant independent predictors of future coronary events in nonculprit lesions.

Our study revealed that NSTE-ACS patients had lesions with significantly negative remodeling index as compared to patients with stable angina, which was consistent with the results of Koo et al. [27] and Fernandes et al. [28]. Conversely, another study showed that all lesions derived from or related to plaque rupture show positive remodeling, which may represent an important surrogate for detecting lesion vulnerability [29].

Our results revealed that SYNTAX score and core composition, especially calcification and lipid composition, were significant predictors for NSTE-ACS. This was different to a study by Zheng et al. [30], in which a multivariate logistic regression model showed that independent predictors for plaque rupture included plaque burden, vessel area, and calcium, while Fujii et al. [31] analyzed 80 plaque ruptures in 74 patients with ACS and showed that independent predictors of culprit plaque ruptures in ACS patients were smaller minimum lumen areas and presence of thrombus.

The plaque vulnerability hypothesis was used to better describe the unpredictability of the future course of atherosclerosis. Vulnerable plaques have been defined as those plaques prone to becoming culprit plaques causing acute coronary events, regardless of stenosis, shape, or destabilization, without taking into account the effect of exogenous factors, such as shear stresses, blood laminar or turbulent flow characteristics, and vascular anatomy and function (e.g., bifurcation and tone, respectively) [8].

Finally, coronary lesions, even with less plaque burden, should not be underestimated because they could be vulnerable plaques and predispose to future cardiac events. IVUS helps to detect plaques morphology and vulnerability, but the PREDICTION study [32] only demonstrated an ability for IVUS and shear stress features to predict plaque enlargement and lumen narrowing without predicting acute events.

The clinical applicability of vulnerable plaques concept has led to advances in our understanding of pathogenesis and management of atherosclerosis. Patients with vulnerable nonculprit lesions require systemic approach rather than localized treatment. Intensifying medical treatment and life-style change may reduce the atherosclerosis burden and future cardiac events.

## 6. Study Limitations

The current study was a single-center study with a small number of patients included.

## 7. Conclusion

The vulnerability rather than the stenotic severity is the most important factor that predispose to Non ST segment elevation acute coronary syndromes. The vulnerability is related to the lesion characteristics especially the lipidic core and calcification while lesion fibrosis favors lesion stability.

## Figures and Tables

**Table 1:** Demographic and clinical data of stable angina and NSTE-ACS groups.

Patients characteristics	All (*n*=116)	NSTE-ACS group (I) (*n*=66)	SA group (II) (*n*=50)	*P* value
Age (years)	52.2 ± 9.2	52.06 ± 9.15	52.05 ± 9.49	0.56

Gender				
Males	90 (77.5%)	48 (72.7%)	42 (84%)	0.18
Females	26 (22.5%)	18 (27.2%)	8 (16%)	0.11

Risk factors				
Hypertension	76 (65.5%)	44 (66.6%)	32 (64%)	0.45
Smoking	74 (63.8%)	42 (63.6%)	32 (64%)	0.56
Diabetes mellitus	50 (43.1%)	30 (45.5%)	20 (40%)	0.34
Dyslipidemia	68 (58.6%)	38 (57.6%)	30 (60%)	0.47
BMI	26.2 ± 3.4	24.8 ± 3.3	27.9 ± 2.9	0.01
FH-IHD	38 (32.7%)	20 (30.3%)	18 (36%)	0.32

Laboratory results				
Total cholesterol (mg%)	183.1 ± 33.03	173.33 ± 29.8	175.73 ± 27.3	0.73
LDL-C (mg%)	132.7 ± 31.48	126.7 ± 36.4	118.86 ± 23.9	0.29
TGL (mg%)	132.8 ± 66.9	149.6 ± 52.6	123.2 ± 32.9	0.045
Previous MI	26 (22.4%)	20 (30.3%)	6 (12%)	0.016
Previous PCI	44 (37.9%)	34 (51.5%)	10 (20%)	0.001
EF (%)	59.03 ± 7.8	59.2 ± 9.1	58.9 ± 6.3	0.21

**Table 2:** Angiographic data of stable angina and NSTE-ACS groups.

Angiographic criteria	All (*n*=312)	NSTE-ACS group (I) (*n*=216)	SA group (II) (*n*=96)	*P* value
Syntax score	12.46 ± 7.6	18.7 ± 7.8	8.07 ± 2.5	0.001

Affected vessel				
LM	21 (6.73%)	18 (8.3%)	3 (3.1%)	0.04
LAD	136 (43.6%)	92 (42.6%)	44 (45.8%)	
CX	74 (23.7%)	49 (22.7%)	25 (26.1%)	0.15
RCA	81 (25.9%)	57 (26.4%)	24 (25%)	

Site of lesion				
Proximal	158 (50.6%)	113 (52.3%)	45 (46.9%)	
Mid	106 (33.9%)	69 (31.9%)	37 (38.5%)	0.24
Distal	48 (15.4%)	34 (15.7%)	14 (14.6%)	

Stenting	134 (42.9%)	80 (37.04%)	54 (56.3%)	0.001

Predilatation	64 (20.5%)	34 (15.7%)	30 (31.3%)	0.001

Edge dissection	2 (0.6%)	2 (0.9%)	0	0.62
Thrombus migration	2 (0.6%)	2 (0.9%)	0	0.62
In-hospital mortality	2 (0.6%)	2 (0.9%)	0	0.62

LM: left main artery; LAD: left anterior descending artery; CX: circumflex artery; RCA: right coronary artery.

**Table 3:** Intravascular ultrasound criteria of SA and NSTE-ACS groups.

IVUS criteria	NSTE-ACS group	Stable angina group	*P* value
Vessel area (EEM CSA) (mm^2^)	9.86 ± 3.8	11.36 ± 2.9	0.001
Max. vessel diameter (mm)	3.76 ± 0.71	4 ± 0.53	0.01
Min. vessel diameter (mm)	3.23 ± 0.66	3.5 ± 0.51	0.01
Lesion MLA (mm^2^)	4.69 ± 2.58	3.58 ± 1.23	0.001
Lesion max. diameter (mm)	2.54 ± 0.62	2.26 ± 0.45	0.001
Lesion min. diameter (mm)	2.05 ± 0.50	1.80 ± 0.38	0.001
Area stenosis (%)	54.7 ± 14.9	68.6 ± 8.7	0.001
Plaque plus media (mm^2^)	5.3 ± 2.54	7.7 ± 2.48	0.001
Plaque burden	54.4 ± 14.7	67.8 ± 9.8	0.001
Lesion length (mm)	17.53 ± 7.3	18.02 ± 9.6	0.68
Proximal reference area (mm^2^)	11.19 ± 3.8	12.83 ± 3.35	0.05
Proximal max. RVD (mm)	4.19 ± 0.47	4.24 ± 0.55	0.69
Proximal min. RVD (mm)	3.79 ± 0.52	3.72 ± 0.62	0.58
Distal reference area (mm^2^)	9.03 ± 3.5	8.79 ± 2.9	0.65
Distal max. RVD (mm)	3.46 ± 0.54	3.63 ± 0.62	0.19
Distal min. RVD (mm)	3.09 ± 0.56	3.30 ± 0.56	0.09
Mean reference area (mm^2^)	9.94 ± 3.5	11.33 ± 3.08	0.01
Remodeling index	0.95 ± 20	1.02 ± 0.14	0.008

EEM: external elastic membrane; CSA: cross-sectional area; MLA: minimal lumen area; RVD: reference vessel diameter.

**Table 4:** Intravascular ultrasound characteristics of coronary lesions.

Lesions structure	NSTE-ACS group	Stable angina group	*P* value
Fibrosis (%)	51.67 ± 7.07	70.37 ± 11.7	0.001
Lipidosis (%)	21.8 ± 7.03	7.26 ± 3.47	0.001
Necrosis (%)	18.08 ± 10.19	15.83 ± 4.9	0.02
Calcification (%)	10.4 ± 5.2	4.19 ± 3.29	0.001

**Table 5:** Predictors of NSTE-ACS.

	*P* value	Odds ratio	95% CI for odds ratio	
			Lower	Upper
Syntax score	0.014	1.289	1.053	1.578
Calcific core percentage	0.002	1.277	1.094	1.491
Necrotic core percentage	0.053	1.089	0.999	1.187
Lipid core percentage	0.001	1.320	1.167	1.494
Vessel CSA	0.538	0.946	0.791	1.130
Plaque burden	0.240	0.951	0.874	1.034

## Data Availability

The data used to support the findings of this study have not been made available because of the hospital policy in respecting patients' privacy.
